# Galectin-1: A Potential Biomarker Differentiating between Early Rheumatoid Arthritis and Spondyloarthritis

**DOI:** 10.3390/jcm11216313

**Published:** 2022-10-26

**Authors:** Ana Triguero-Martínez, Emilia Roy-Vallejo, Eva Gloria Tomero, Nuria Montes, Hortensia de la Fuente, Ana María Ortiz, Santos Castañeda, Amalia Lamana, Isidoro González-Álvaro

**Affiliations:** 1 Rheumatology Department, Hospital Universitario La Princesa, Instituto de Investigación Sanitaria La Princesa (IIS-IP), 28006 Madrid, Spain; 2 Internal Medicine Department, Hospital Universitario La Princesa, Instituto de Investigación Sanitaria La Princesa (IIS-IP), 28006 Madrid, Spain; 3 Immunology Department, Hospital Universitario La Princesa, Instituto de Investigación Sanitaria La Princesa (IIS-IP), 28006 Madrid, Spain; 4 Cell Biology Department, Facultad de Biología, Universidad Complutense de Madrid, 28040 Madrid, Spain

**Keywords:** spondyloarthritis, Galectin-1, biomarker, rheumatoid arthritis, early arthritis

## Abstract

Galectin-1 (Gal1) plays a regulatory role in the immune system. We have recently validated that Gal1 serum (sGal1) levels are increased in rheumatoid arthritis (RA) patients compared to healthy donors (HDs); however, there is no information on Gal1 in spondyloarthritis (SpA). **Objective:** To compare Gal1 levels in patients with SpA versus RA as a diagnostic biomarker. **Methods:** We studied sGal1 levels in HD (n = 52), SpA (n = 80) and RA patients (n = 64) who were randomly divided into discovery and validation sets. Synovial fluid (SF) from osteoarthritis (OA) (n = 28), peripheral SpA (n = 28) and RA (n = 28) were studied. In SpA patients, we analyzed the association between clinical parameters and sGal1 levels. **Results**: sGal1 levels were significantly lower in patients with SpA with respect to RA and similar to those of the HD. A cut-off of 20.50 ng/mL (sGal1) allowed one to differentiate RA patients from SpA and HD (Odd Ratio (OR) 8.23 and 12.64, respectively). Gal1 SF levels in SpA were slightly lower than OA patients and significantly lower than RA patients. No correlation was observed between sGal1 levels and clinical parameters in SpA patients. **Conclusion:** Gal1 could act as a diagnostic biomarker of RA and would allow one to distinguish SpA and RA patients.

## 1. Introduction

Immune-mediated inflammatory diseases (IMIDs) are a broad group of diseases, including psoriasis, rheumatoid arthritis (RA), inflammatory bowel disease, ankylosing spondylitis (AS) and connective tissue diseases (CTDs), among others, that can cause peripheral arthritis. IMIDs share pathogenic mechanisms involving components of adaptive and innate immunity [1,2].

Spondyloarthritis (SpA) is the second most frequent group of IMID-causing arthritis (prevalence between 0.4 and 1.9%) [3,4]. Early diagnosis of SpA is challenging due to the absence of pathognomonic features, which may lead to a delay in treatment, causing a large symptomatic burden and, as a consequence, functional disability during the most productive years of working life [5].

RA is the most common IMID-causing arthritis. It is considered a multifactorial disease in which the interaction between genetic, environmental and social factors leads to a breakdown of self-recognition, resulting in chronic infiltration of the synovial membrane by a wide variety of immune cells and exacerbation of proinflammatory cytokine responses [6]. In absence of treatment, it leads to joint destruction due to cartilage degradation and bone erosion [7,8].

There is strong evidence supporting that early detection of an IMID and prescription of immunomodulatory treatment within the first year after the onset of symptoms provide a better chance of good response and a greater possibility of reaching remission. This has been clearly established for RA [9,10] and there is some evidence indicating that it could also be true for other IMIDs [11,12]. In this regard, during the last two decades early arthritis clinics have been established in many clinical settings all around the world. These clinics have improved research in RA and SpA, as well as the implementation of early treatment in these patients [13]. Nevertheless, the early recruitment of patients leads to the classification of a higher percentage of cases as undifferentiated arthritis (UA); these patients usually receive methotrexate as the initial treatment. However, subsequent immunomodulatory treatments may be based on different mechanisms such as cytokine blockade (for TNF, IL-6, IL-17, IL-12/23 and B lymphocyte stimulator (BlyS)), B-cell depletion or costimulation blockade, depending on the type of underlying IMID. Therefore, to improve therapeutic choice, additional diagnostic biomarkers are needed to better classify patients with early arthritis, especially those not fulfilling specific classification criteria.

Gal1 belongs to the lectin family of proteins that bind β-galactoside carbohydrates [14,15]. It might be involved in RA pathogenesis [16] since it is highly expressed in immune cells [17] acting as a novel regulatory checkpoint that links innate and adaptive responses [18]. Recently, we confirmed that patients with RA present increased Gal1 serum level (sGal1) [19,20]. However, to date there is no solid information on the role that Gal1 may play in SpA. Therefore, the objective of this work was to determine potential differences between sGal1 levels in SpA patients, RA patients and healthy donors (HDs) in order to determine whether this molecule could be useful for differentiating between SpA and RA, especially in early phases in which many patients appear as undifferentiated arthritis.

## 2. Patients and Methods

For this study, 52 HD, 80 SpA and 64 RA patients were randomly assigned to population 1 (discovery phase) and population 2 (validation phase). The different study populations are described below. 

### 2.1. Spondyloarthritis Population 

The 80 spondyloarthritis patients belonged to different subpopulations:

#### 2.1.1. Early Spondyloarthritis Population 

This subpopulation comprised 31 early SpA patients (population 1 n = 15; population 2 n = 16) from the PrincEsa Register of Spondyloarthritis with Early Onset (PERSEO). PERSEO protocol includes seven prospective visits (baseline, 1, 2, 4, 6, 8 and 10 years). However, clinical data and samples obtained from the first four years of follow-up (a total 76 visits) were analyzed only in 19 patients just to confirm that sGal1 levels do not significantly change along the follow-up as described previously in RA patients [20]. In the remaining 12 patients, sGal1 data and clinical data corresponded to the baseline visit.

Previously described PERSEO inclusion criteria are inflammatory back pain (IBP) for more than 3 months and less than 2 years, and symptom onset before the age of 45 [21]. Socio-demographic, clinical information including assessment of disease activity by Bath Ankylosing Spondylitis Disease Activity Index (BASDAI) and Ankylosing Spondylitis Disease Activity Score with C-Reactive Protein (ASDAS-CRP) and disability by Bath Ankylosing Spondylitis Functional Index (BASFI), therapeutic and laboratory data are recorded and included in an electronic database. Biological samples are collected at each visit and stored at −80 °C at the Instituto de Investigación Sanitaria La Princesa (IIS-IP) Biobank. At each visit during the follow-up, patients of PERSEO are checked for fulfilment of the Assessment of SpondyloArthritis International Society (ASAS) criteria for SpA [22]. All patients included in the study fulfilled ASAS criteria for SpA; patients with other diagnoses were excluded from the study except those who finally were classified as mechanical lower back pain patients, who were included in the control population group. 

#### 2.1.2. Long Term Ankylosing Spondylitis Population 

The second SpA subpopulation comprised 28 patients (population 1 n = 14; population 2 n = 14) with long term ankylosing spondyloarthritis (AS) assessed in a single cross-sectional visit. All patients fulfilled the New York classification criteria for ankylosing spondylitis [23]. In order to homogenize variables, the information collected in this visit was the same as that collected in early SpA. Biological samples were also collected and stored as described for PERSEO.

#### 2.1.3. Psoriatic Arthritis

Finally, the third SpA subpopulation comprised 21 psoriatic arthritis (PsA) patients (population 1 n = 10; population 2 n = 11) from the Princesa Early Arthritis Register Longitudinal (PEARL) study, who fulfilled ClASsification criteria for Psoriatic Arthritis (CASPAR) [24]. A more detailed description of PEARL protocol is provided below.

### 2.2. Early Rheumatoid Arthritis Population 

Sixty-four patients (population 1 n = 32; population 2 n = 32) from the PEARL study who fulfilled 1987 ACR classification criteria [25] after two years of follow-up were used as RA group to be compared with SpA.

The PEARL study comprises patients with one or more swollen joints and symptoms with ≤ 1 year of evolution. The register protocol includes 4 visits during a 2-year follow-up (0, 6, 12 and 24 months). Socio-demographic, clinical, therapeutic and laboratory data are recorded and included in an electronic database. Biological samples are collected at each visit and stored at −80 °C at the IIS-IP Biobank for translational research. A more detailed description of PEARL protocol has been previously published [26]. 

### 2.3. Healthy Donors

Serum samples from 52 healthy donors (population 1 n = 26; population 2 n = 26) were included in this study, of whom 24 were mechanical lower back pain patients from the PERSEO population, 20 were patients from the PEARL population in which the presence of arthritis or any autoimmune disorder was excluded, and the remaining controls were obtained from the IIS-IP Biobank.

### 2.4. Synovial Fluid Samples 

Synovial fluid (SF) samples were obtained from therapeutic or diagnostic knee arthrocentesis. Since the samples were anonymized, only information about diagnosis was available. Hematic samples were discarded.

Samples were centrifuged at 2000× *g* rpm during 20 min at room temperature and the cell-free supernatants were collected and stored at −80 °C until analysis. For this study, samples from 28 OA patients, 28 peripheral SpA patients and 28 RA patients were analyzed. These samples were obtained from different patients than those providing serum samples. We used OA SF as a non-autoimmune inflammatory control group.

### 2.5. Measurement of Serum and Synovial Fluid Gal1 

We use Quantikine Human Gal1 Immunoassay (R&D Systems, Minneapolis, MN, USA) to measure Gal1 serum and SF levels according to the manufacturer’s instructions. Absorbance was measured in a spectrophotometer (Innogenetics Diagnostica y terapeutica S.A.U, Barcelona, Spain) at 450 nm with correction at 620 nm. Measurements for all samples were performed in duplicate. In order to minimize the influence of inter-assay variability, in every plate we included samples from the different subgroups of the study.

### 2.6. Ethics Approval and Consent to Participate

The present study was approved by the Research Ethics Committee of Hospital Universitario de La Princesa (PI-467; 9 September 2010, PI-518; 28 March 2011) and it complied with principles expressed in the Helsinki Declaration of 1983 and successive actualizations. To participate in the study it was necessary to sign a written informed consent and to be older than 18 years old. The IIS-IP Biobank (ISCIII B.0000763) provided the samples and data from patients included in this study. All samples were processed following standard operating procedures with the appropriate approval of the Ethics and Scientific Committees.

### 2.7. Statistical Analysis

Statistical analyses were performed using Stata 14.0 for Windows (Stata Corp LP, College Station, TX, USA). Most quantitative variables followed a non-normal distribution, so they were represented as median and interquartile range (IQR), and the Mann–Whitney or Kruskal–Wallis tests were used to analyze significant differences. Qualitative variables were described as proportions, and the χ2 or Fisher’s exact test was used to compare categorical variables. The Spearman´s *rho* test was applied to analyze correlation between quantitative variables. *p*-values below 0.05 were considered statistically significant.

Considering that sGal1 levels significantly increased with age, to achieve a more accurate graphical representation, we obtained age-adjusted values of sGal1 using the command “margins” of Stata once a multivariable analysis was fitted including age and sex as independent variables.

Finally, to assess the ability of sGal1 levels to discriminate between SpA and RA patients, using the data from population 1, we performed Receiver Operating Characteristic (ROC) curve analysis through the command roctab, comparing the data of sGal1 levels in patients with SpA with the sGal1 levels in patients with RA. For development of ROC curves, the option graph was used. The cut-off point was selected on the basis of the best trade-off values between sensitivity, specificity, cases correctly classified and positive and negative likelihood ratios (LR+ and LR−, respectively) obtained using the command roctab with the option detail. 

To validated this cut-off value in population 2 we fitted a logistic regression with the dependent variable being sGal1 levels as the dichotomic variable (low if below the cut-off vs high if above the cut-off) and adjusting the model by sex and age, and including also as independent variable the diagnosis (HD, SpA and RA).

## 3. Results

### 3.1. Gal1 Serum Levels Remain Stable along the Follow-Up and Are Not Associated with Disease Activity or Disability in Early SpA

**Figure 1:**
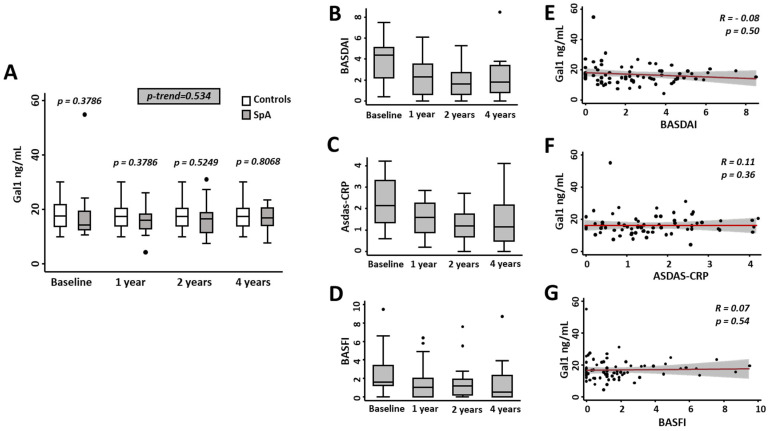
Gal1 serum levels do not correlate with clinical severity parameters in early spondyloarthritis. (**A**) Determination of Gal1 serum levels with ELISA in control (mechanical lower back pain patients) and early spondyloarthritis (SpA) patients during the follow-up. Gal1 serum levels were adjusted by age as described in the methods section. Statistical significance for the trend of Gal1 in the follow-up visits in early SpA patients was determined with Cuzick’s non-parametric test. Significance threshold was set at *p*-trend < 0.05. (**B**) Evolution along the follow-up of disease activity was assessed with BASDAI or (**C**) ASDAS-CRP, and disability was assessed with BASFI (**D**) in early spondyloarthritis patients. Absence of correlation of Gal1 serum levels with BASDAI (**E**), ASDAS-CRP (**F**) and BASFI (**G**) in early spondyloarthritis patients. In panels A to D, data are shown as interquartile range (p75 upper edge of box, p25 lower edge, p50 midline) as well as p95 (line above box) and p5 (line below). Dots represent outliers. In panels E to G, data are shown as dot plots and their fitted linear prediction with 95% confidence interval (grey shadow) using the two-way command of Stata with the lfitci option. Spearman’s *rho* test was used to determine the level of significance.

First, considering that our previous study with RA patients showed that sGal1 levels remain stable irrespective of disease activity [20], we analyzed samples from 19 early SpA and 24 mechanical lower back pain patients (clinical characteristics in Supplementary Table S1) from our longitudinal register PERSEO. As shown in Figure 1A, there were no significant differences in sGal1 levels between both groups at any time point, nor were significant differences observed in the follow-up in early SpA patients. Thus, we validated our previous observation in RA, suggesting that sGal1 levels remain stable in the follow up and it would be valid to measure Gal1 concentration at any point in time to check its value as a diagnostic biomarker.

As described for RA, despite improvement of disease activity assessed with BASDAI (Figure 1B) or ASDAS-CRP (Figure 1C) and disability evaluated with BASFI (Figure 1D), no significant correlation was observed between sGal1 levels and BASDAI, ASDAS or BASFI (Figure 1E–G).

### 3.2. Study Population

Then, considering the previous information, we performed a cross-sectional study with a total of 80 SpA patients (early spondyloarthritis n = 31; psoriatic arthritis n = 21; ankylosing spondylitis n = 28), 64 RA patients and 52 HDs that were split into two populations as described in the patients and methods section. The clinical characteristics and treatments of the different populations are shown in Table 1, Tables S2 and S3.

SpA patients were significantly younger, had longer disease duration at the beginning of the follow-up and a higher frequency of males compared to RA patients. Due to these differences, we analyzed whether variables such as sex or age influenced sGal1 levels. sGal1 levels significantly increased with age, whereas no significant differences were observed by sex (Supplementary Figure S1). Therefore, age was taken into consideration in subsequent analyses of sGal1 levels.

### 3.3. Gal1 Serum Levels Are Similar in HD and in SpA Patients but LOWER than in Early RA Patients

#### 3.3.1. Discovery Phase (Population 1)

**Figure 2:**
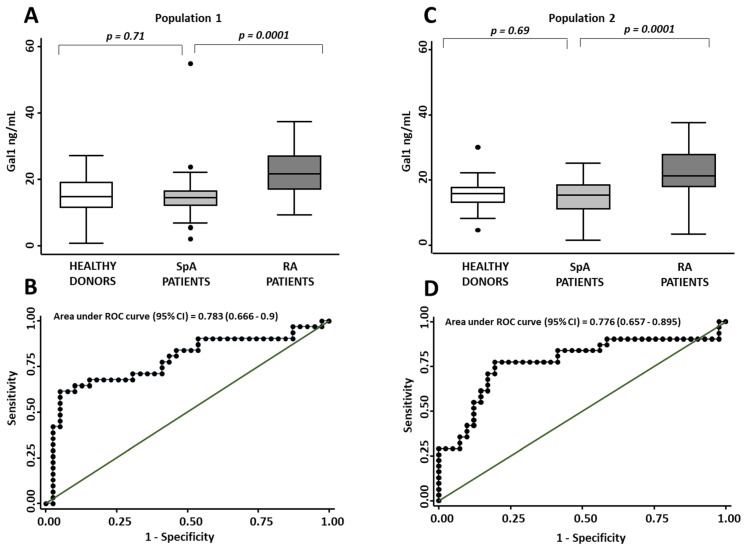
Spondyloarthritis patients and healthy donors show similar Gal1 serum levels and lower Gal1 serum levels than those in early rheumatoid arthritis patients. (**A**,**C**) determination of Gal1 serum levels with ELISA in healthy donors, spondyloarthritis (SpA) patients and early rheumatoid arthritis (RA) from populations 1 and 2, respectively. Gal1 serum levels were adjusted by age as described in the methods section. Data are shown as interquartile range (p75 upper edge of box, p25 lower edge, p50 midline) as well as p95 (line above box) and p5 (line below). Dots represent individual values. Statistical significance was determined with the Kruskal–Wallis test. Significance threshold was set at *p* < 0.05. (**B**,**D**) ROC curve analysis to assess Gal1 capacity to discriminate between SpA patients and RA patients in populations 1 and 2, respectively.

sGal1 levels were not significantly different in HD and SpA patients (*p* = 0.71, Figure 2A); however, the sGal1 levels in both populations were significantly lower than those of RA patients (*p* < 0.001, Figure 2A). Even after adjustment by sex and age, the differences by diagnosis remained significant (Table 2). Furthermore, when disease-modifying anti-rheumatic drugs were forced in the model, none of them showed a relevant effect on sGal1 levels (data no shown). In addition, no significant differences were found in sGal1 levels between the different subgroups of SpA (early SpA, AS, PsA) and HDs, but they were significantly lower than those of RA patients (Supplementary Figure S2 and Supplementary Table S4).

In view of these results, the next step was to determine the capability of sGal1 levels to discriminate between RA and SpA patients. The ROC analysis (AUC = 0.783; confidence interval (CI) 95% (0.666–0.9)) showed that a Gal1 concentration above 20.50 ng/mL had 67.74% sensitivity (LR− 0.40) and 79.49 % specificity (LR+ 3.30) in terms of differentiating those diagnoses (Figure 2B). 

#### 3.3.2. Validation Phase (Population 2)

Data from population 2 confirmed that sGal1 levels were similar in HD and SpA patients (*p* = 0.69, Figure 2C) and both were significantly lower than those of RA patients (*p* < 0.001, Figure 2C).

In population 2, the area under the ROC curve was very close to that of population 1 (0.776 (CI 95% 0.657–0.895) vs 0.783 (0.666–0.9); Figure 2B,D) and the cut-off point selected in population 1 (20.5 ng/mL) rendered 77.4% sensitivity (LR−0.29) and 75.6% specificity (LR+ 3.17) in population 2.

**Figure 3:**
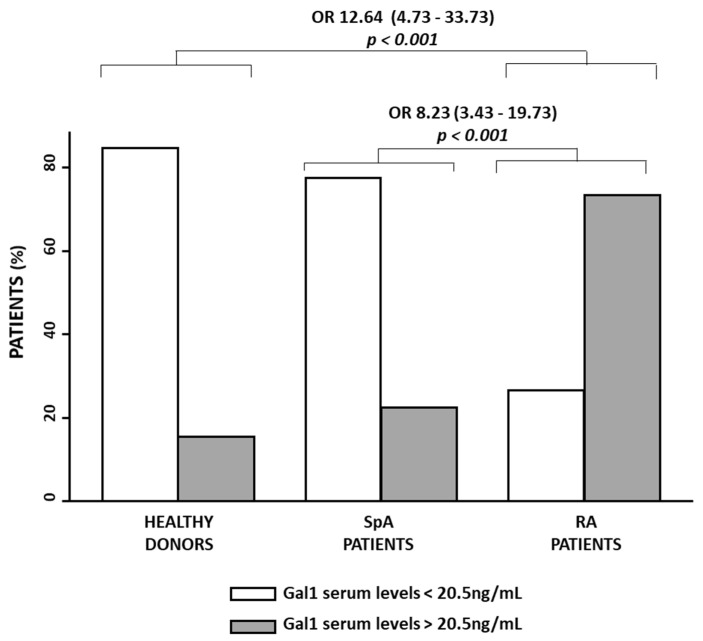
Gal1 serum levels could differentiate between rheumatoid arthritis patients and spondyloarthritis patients or healthy donors. Comparison of the percentage of patients with low Galectin-1 (Gal1) serum levels (white bar) versus patients with high Gal1 serum levels (grey bar) in the different study subpopulations. The cut-off for discriminating high and low Gal1 serum levels was 20.5 ng/mL, as described in the results section. Odd Ratio (OR) and its Confidence Interval (CI) were estimated with the logit command of Stata 14.1. The model was adjusted by sex and age. Significance threshold was set at *p*-value < 0.05.

In view of these validation results, we calculated the percentage of patients of the whole population whose sGal1 concentration was above 20.5 ng/mL. Furthermore, we performed a multivariable logistic regression analysis to calculate the Odd Ratio (OR) for being RA when sGal1 levels are high in comparison to SpA patient (OR 8.23 (CI 95% 3.43–19.73)), *p* < 0.001) or HD (OR 12.64 (CI 95% 4.73–33.73)), *p* < 0.001) (Figure 3). 

### 3.4. Gal1 Synovial Fluid Levels Are Lower in Peripheral SpA Compared to OA Patients

**Figure 4:**
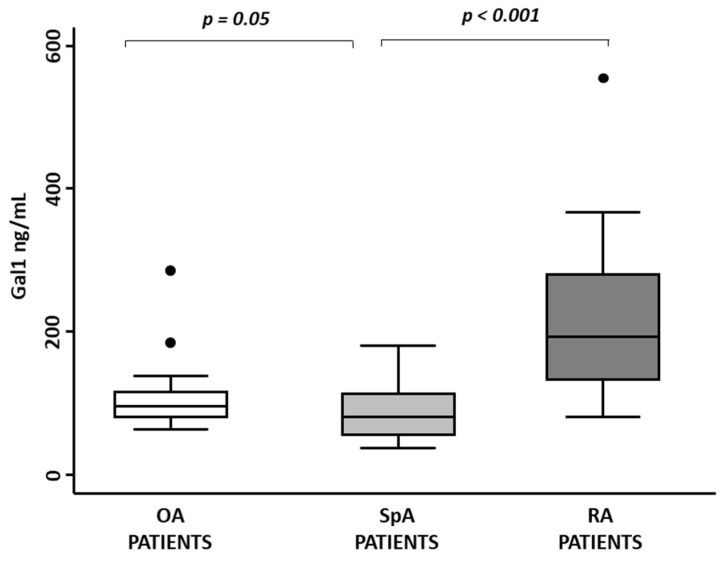
Gal1 synovial fluid levels are different in distinct arthritis populations. Determination of Gal1 synovial fluid levels with ELISA in osteoarthritis (OA) patients, spondyloarthritis (SpA) patients and rheumatoid arthritis (RA) patients. Data are shown as interquartile range (p75 upper edge of box, p25 lower edge, p50 midline) as well as p95 (line above box) and p5 (line below). Dots represent individual values. Statistical significance was determined with ANOVA. Significance threshold was set at *p*-value < 0.05.

Finally, in order to confirm the previous results locally at the joint compartment, we compared Gal1 levels in SF of 28 patients with OA, 28 with peripheral SpA and 28 with RA. We observed that SpA patients had slightly lower Gal1 SF levels than OA patients (*p* = 0.05, Figure 4). However, RA patients had significantly higher Gal1 levels than OA or SpA patients (*p* < 0.001, Figure 4).

## 4. Discussion

One of the main concerns in the management of IMIDs is early detection and diagnosis in order to treat them as soon as possible since this ensures a higher probability of achieving complete remission. Nonetheless, early phases of IMIDs frequently preclude from making a precise diagnosis, so additional biomarkers are needed. In this scenario, several studies have been reported and it was validated that sGal1 levels are increased in RA patients compared to healthy donors [19,20]. However, as far as we know, Gal1 has not been previously studied in SpA. This approach is needed to verify whether increased levels of Gal1 are specific to RA or a common finding for different IMIDs. So, the main result of our work is that Gal1 levels in both serum and SF are significantly lower in patients with SpA compared to RA patients. Interestingly, sGal-1 levels were also able to differentiate patients with SpA from those with RA. All in all, these results raise the possibility that measurement of Gal1 in biological fluids could be a useful diagnostic tool.

During recent decades, it has been shown that IMIDs share immunopathogenic mechanisms, resulting in inflammation frequently located at joints. Thus, different IMIDs can present, at the beginning of the disease, with similar symptoms at articular level. Although some disease-modifying antirheumatic drugs (DMARDs) such as methotrexate, sulfasalazine or leflunomide can be useful for the treatment of peripheral arthritis, there are subtle differences in its response to these drugs, and especially to biological treatments [22,27]. Therefore, additional diagnostic biomarkers are needed in order to better classify patients with peripheral involvement (e.g., peripheral arthritis, enthesopathy or dactylitis) not fulfilling specific classification criteria [28]. Psoriatic arthritis can be a paradigmatic example for this situation due to its characteristic absence of autoantibodies and rare HLA-B27 positivity. Our data show that patients with PsA show lower Gal1 levels in both serum and SF compared to RA.

Furthermore, considering SpA as a whole group, an sGal1 level higher than 20.5 ng/mL showed a good performance in terms of differentiating SpA patients from those with RA (OR 8.23 (CI 95% 3.43–19.73)); *p* < 0.001). This cut-off is very similar to that proposed in our previous study in which an sGal1 level higher than 19 ng/mL differentiated properly RA patients and HDs [17]. The differences between both studies could be due to the inter-assay variation coefficient (8.5% approximately according to the ELISA kit manufacturer). 

Interestingly, a subpopulation of the current study is included in a longitudinal cohort of patients with early SpA (PERSEO). As it happened with the longitudinal study of early arthritis (PEARL) [20], no relevant variation in sGal1 levels was observed across the follow-up in PERSEO, suggesting that sGal1 levels are not altered by treatment or disease duration. Therefore, measurement of sGal1 levels could be useful as a diagnostic biomarker at any time during the course of the disease.

On the other hand, using Gal1 as a diagnostic biomarker has some limitations. One of them, described in this work, is the increase of sGal1 levels with age. Other authors have already reported an increase in this protein with age and even proposed a role for endogenous Gal1 as a key immune regulator that promotes immune tolerance and prevents age-dependent spontaneous autoimmunity [29]. However, the increase with age raises the question of whether different cut-offs would be necessary depending on age. Another possible limitation is the sample size and heterogeneity of the study population, especially because it was composed of three different SpA subpopulations. However, despite the atomization involved in dividing the samples into two populations, the sGal1 levels are very similar, which demonstrates the consistency of the data and provides robustness to the study. Nevertheless, validation studies in other SpA populations, as well as characterization of sGal1 levels in CTD such as systemic lupus erythematosus, systemic sclerosis or Sjögren syndrome are needed.

In conclusion, the principal aim of this study was to evaluate the diagnostic value of Gal1 in SpA patients and compare the results with previously studies in other IMIDs that cause arthritis such as AR. The findings of this work suggest that sGal1 levels are similar between HDs and SpA patients and do not correlate with clinical severity parameters. However, this suggests the possibility that Gal1 could be a possible diagnostic biomarker to differentiate between SpA and RA patients when the patients are at early stages of the disease, especially in those patients HLA-B27, RF and ACPA negative. In this regard, it would be an interesting in an additional study to test in patients classified initially as undifferentated arthritis whether baseline Gal1 serum levels help to differentiate RA and SpA patients.

## Figures and Tables

**Table 1:** Baseline characteristics of the populations studied.

	Healthy Donors			SpA Patients			RA Patients		
	Population 1	Population 2	*p*-Value	Population 1	Population 2	*p*-Value	Population 1	Population 2	*p*-Value
	(n = 26)	(n = 26)		(n = 39)	(n = 41)		(n = 32)	(n = 32)	
Female; n (%)	17 (65.4)	15 (57.7)	0.8	17 (47.2)	16 (40.0)	0.6	28 (87.5)	24 (75)	0.3
Age; p50 [p25–p75]	38 (32.2–63)	33.6 (31.6–49.7)	0.1	48.5 (32.5–56.9)	48.48 (34–57.3)	0.7	54 (44.9–67.3)	56.21 (41.4–68.8)	0.8
Disease duration (months); p50 [p25–p75]	-	-	-	13 (5.53–204)	18 (6–276)	0.7	4.6 (2.4–9.1)	5.3 (1.9–9.6)	0.9
HLA-B27 positive; n (%)	-	-	-	21 (72.4)	24 (68.6)	0.7	-	-	-
RF positive; n(%)	-	-	-	-	-	-	20 (62.5)	24 (75)	0.4
ACPA positive; n(%)	-	-	-	-	-	-	18 (56.3)	20 (62.5)	0.4
BASDAI; p50 [p25–p75]	-	-	-	3 (2–4.5)	3.5 (1.5–5)	0.9	-	-	-
BASFI ; p50 [p25–p75]	-	-	-	2.6 (1.2–3.2)	1.65 (1.2–2.6)	0.4	-	-	-
ASDAS ; p50 [p25–p75]	-	-	-	2.5 (1.9–3.2)	1.88 (1.4–3)	0.2	-	-	-
DAS28; p50 [p25–p75]	-	-	-	-	-	-	4.79 (3.2–5.8)	4.5 (3.5–5.8)	0.5
HAQ; p50 [p25–p75]	-	-	-	-	-	-	1 (0.5–1.4)	1 (0.6–1.6)	0.9

n: number; p50: median or percentile 50; p25–p75: range between percentiles 25 and 75 or interquartile range; ACPA: anti-citrullinated protein antibodies; ASDAS: Ankylosing Spondylitis Disease Activity index; BASDAI: Bath Ankylosing Spondylitis Disease Activity Index; BASFI: Bath Ankylosing Spondylitis Functional Index; DAS28: Disease Activity Score estimated with 28 joint count; HAQ: Health Assessment Questionnaire; RA: Rheumatoid Arthritis; SpA: Spondyloarthritis. HLA-B27: Human Leukocyte Antigen B27; RF: Rheumatoid Factor.

**Table 2:** Relationship between Gal1 (ng/mL) serum levels and diagnosis.

	β Coeff. (95% CI)	*p* Value
**Sex**		
Male	Reference	
Female	−1.09 (−3.53–1.35)	0.37
**Age (years)**		
<45	Reference	
45–65	1.16 (−1.40–3.73)	0.372
>65	2.92 (−0.24–6.08)	0.07
**Diagnosis**		
Spondyloarthritis	Reference	
Healthy donors	−0.43 (−3.23–2.36)	0.75
Rheumatoid arthritis	6.54 (3.61 – 9.47)	<0.001

CI: Confidence Interval; Coeff: Coefficient.

## Data Availability

The datasets used and/or analyzed during the current study are available from the corresponding author (isidoro.ga@ser.es) on reasonable request.

## Supplementary Materials

The following supporting information can be downloaded at: https://www.mdpi.com/article/10.3390/jcm11216313/s1, Figure S1: Relationship between Gal1 serum levels and clinical characteristics; Figure S2: Gal1 serum levels in different types of spondyloarthropathies in comparison with healthy donors and rheumatoid arthritis patients; Table S1: Baseline characteristics of the early spondyloarthritis and mechanical low back pain patients population studied; Table S2: Baseline treatment of the populations studied; Table S3: Baseline characteritics of spondyloarthropahies subpopulations; Table S4 :Relationship between Gal1 (ng/ml) serum levels and diagnosis.
