# Supplementary material for: Galectin-1: A Potential Biomarker Differentiating between Early Rheumatoid Arthritis and Spondyloarthritis
Source: J Clin Med. 2022 Oct 26;11(21):6313. doi: 10.3390/jcm11216313 (PMC9658544; doi:10.3390/jcm11216313)
Supplement: Supplementary file 1 [file jcm-11-06313-s001.zip › jcm-1929348-supplementary1.pdf]

Journal of
Clinical Medicine

MDPI

Supplementary material

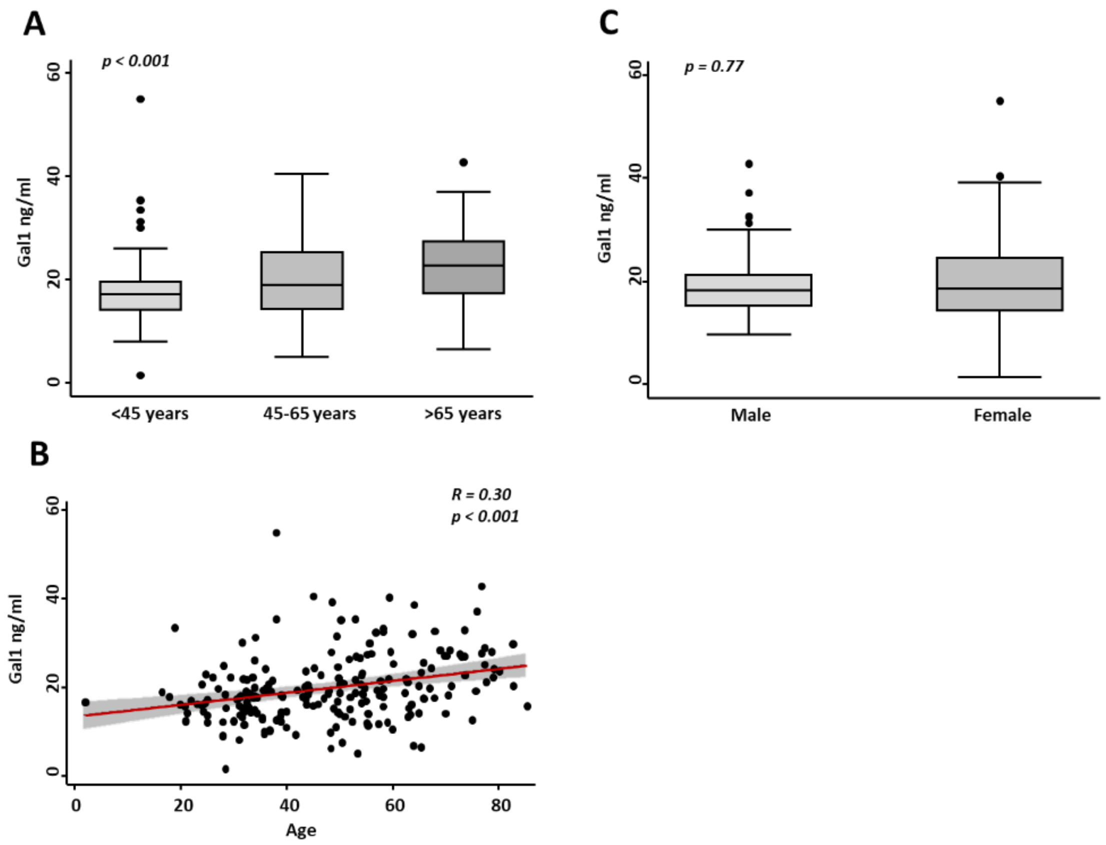

Figure S1. Relationship between Gal1 serum levels and clinical characteristics. Determination of Gal1 serum levels by ELISA in all populations of study and age **(A)** or sex **(C).** Data shown as interquartile range (p75 upper edge of box, p25 lower edge, p50 midline) as well as the p95 (line above box) and p5 (line below). Dots represent outliers. Statistical significance was determined with ANOVA or Kruskal-Wallis test respectively. Significance threshold was set at $p < 0.05$. Correlation of Gal1 serum levels with age in all populations of study **(B)**. Data shown as dot plots and their fitted linear prediction with 95% confidence interval (grey shadow) using the *twoway* command of Stata with the *lfitci* option. Pearson correlation test was used to determine the level of significance.

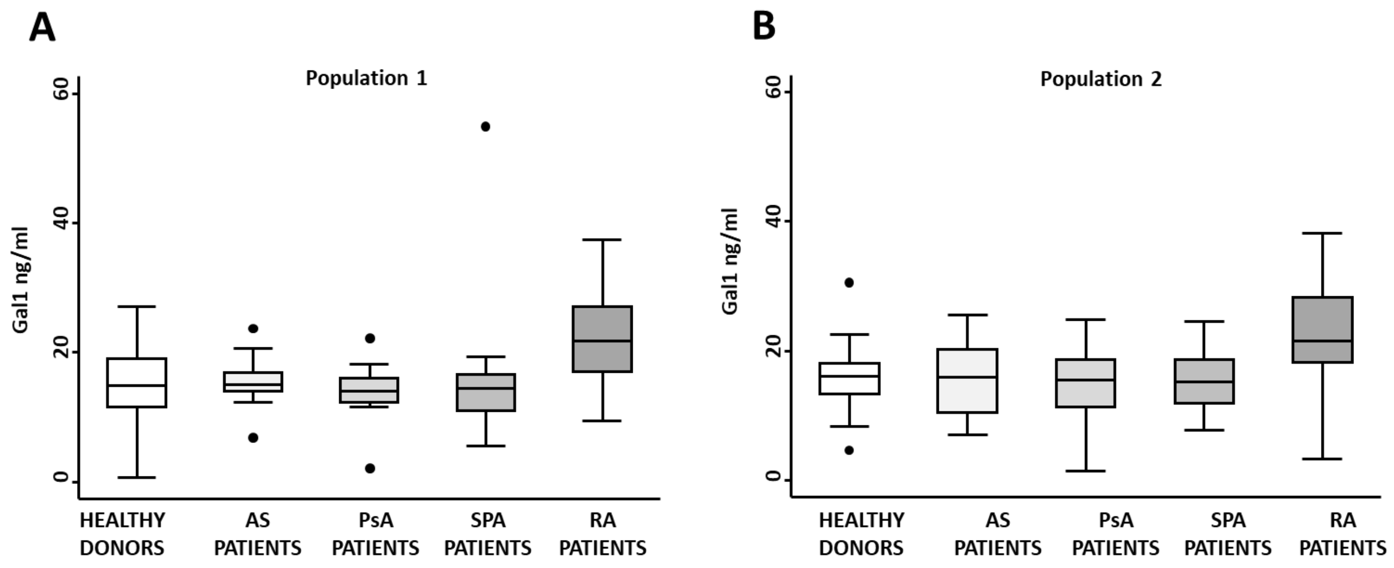

Figure S2. Gal1 serum levels in different types of spondyloarthropathies in comparison with healthy donors and rheumatoid arthritis patients. Determination of Gal1 serum levels by ELISA in healthy donors, ankylosing spondylitis (AS), psoriatic arthritis (PsA) early spondyloarthritis (SPA) and early rheumatoid arthritis (RA) patients in population1 (discovery phase) (**A**) and population 2 (validation phase) (**B**). Gal1 serum levels were adjusted as describe in methods. Data are shown as interquartile range (p75 upper edge of box, p25 lower edge, p50 midline) as well as the p95 (line above box) and p5 (line below). Dots represent outliers. Statistical significance was determined with the Kruskal-Wallis test. Significance threshold was set at $p < 0.05$.

Table S1. Baseline characteristics of the early spondyloarthritis and mechanical low back pain patients population studied.

	**Mechanical low back pain patients** (***n*** **= 24)**	**SPA patients** (***n*** **= 19)**	***p*-value**
Female; *n* (%)	9 (37.5)	12 (63.16)	0.08
Age; p50 [p25-p75]	36.5 [33 – 40.5]	42 [36 – 45]	0.06
Disease duration (months); p50 [p25-p75]		18 [5 – 24]	
HLA-B27 positive; *n* (%)		16 (84.21)	
BASDAI; p50 [p25-p75]		4.4 [2.2 – 5.1]	
BASFI ; p50 [p25-p75]		1.60 [1.2 – 3.4]	
HAQ; p50 [p25-p75]		0.125 [0 – 0.625]	

n: number; p50: median or percentile 50; p25-p75: range between percentiles 25 and 75 or interquartile range; SPA: spondyloarthritis; BASDAI: Bath Ankylosing Spondylitis Disease Activity Index; BASFI: Bath Ankylosing Spondylitis Functional Index HAQ: baseline health assessment questionnaire.

Table S2. Baseline treatment of the populations studied.

	**Early RA patients (*n* = 52)**	**AS patients (*n* = 21)**	**early SpA patients (*n* = 19)**	**Psoriatic arthritis patients (*n* = 15)**
Methotrexate (*n*)	8	3		3
Leflunomide (*n*)		1		1
Hidroxicloroquine (*n*)	2			
Salzopyrine (*n*)		6	9	
Anti-TNF (*n*)		12		

n: number;RA: rheumatoid arthritis; AS: ankylosing spondylitis; SpA: spondyloarthritis;TNF: tumor necrosis factor.

Table S3: Baseline characteritics of spondyloarthropahies subpopulations.

	Population 1				Population 2			
	AS patients	Early SpA patients	psoriatic arthritis patients	*p*-value	AS patients	Early SpA patients	psoriatic arthritis patients	*p*-value
	(*n* = 14)	(*n* = 15)	(*n* = 10)		(*n* = 14)	(*n* = 16)	(*n* = 11)	
Female; *n* (%)	4 (28.57)	7 (58.33)	6 (60)	0.2	2 (15.38)	7 (43.75)	7 (63.64)	0.05
Age; p50 [p25-p75]	53.44 [40 – 74.98]	35 [30 – 49]	48.34 [26.03 – 53.35]	0.05	57.31 [48.31 – 63.3]	39.5 [33 – 53.5]	42.37 [28.47 – 55.2]	0.02
Disease duration (months); p50 [p25-p75]	264 [216 – 348]	11 [5 – 16]	6.8 [4.03 – 13.73]	< 0.001	294 [276 – 360]	18 [10.5 – 24]	4.23 [1.86 – 7]	< 0.001
HLA-B27 positive; *n* (%)	8 (88.89)	11 (91.67)	2 (25)	0.002	14 (100)	12 (75)	0 (0)	< 0.001
BASDAI; p50 [p25-p75]	2.9 [2 – 3.6]	3.85 [1.7 – 5.15]		0.3	3.05 [1.7 – 3.8]	3.9 [1.25 – 5.3]		0.53
BASFI ; p50 [p25-p75]	2.6 [1.5 – 3.1]	2.7 [0.6 – 3.4]		0.91	1.95 [1.5 – 2.6]	1.45 [1.1 – 2.7]		0.42
ASDAS ; p50 [p25-p75]	2.2 [1.9 – 2.5]	3.11 [1.81 – 3.62]		0.13	1.85 [1.6 – 3.05]	1.97 [1.37 – 2.77]		1
DAPSA; p50 [p25-p75]			8.79 [5.49 – 10]				6.9[4.58 – 11.96]	

n: number; p50: median or percentile 50; p25-p75: range between percentiles 25 and 75 or interquartile range; AS: ankylosing spondylitis ; SPA: spondyloarthritis;RA: rheumatoid arthritis; BASDAI: Bath Ankylosing Spondylitis Disease Activity Index; BASFI: Bath Ankylosing Spondylitis Functional Index; ASDAS: Ankylosing spondylitis disease activity index;DAS28: disease activity score estimated with 28 joint count; DAPSA: disease activity psoriatic.

Table S4. Relationship between Gal1 (ng/ml) serum levels and diagnosis.

	Population 1		Population 2	
	β Coeff. (95% CI)	*p*-value	β Coeff. (95% CI)	*p*-value
Female gender	-0.17 (-3.63 to 3.27)	0.91	-1.65 (-4.58 to 1.28)	0.26
**Age (years)**				
< 45	Reference		Reference	
45–65	1.65 (-1.93 to 5.24)	0.36	2.35 (-0.73 to 5.43)	0.13
> 65	3.08 (-0.96 to 7.13)	0.13	3.24 (-1 to 7.49)	0.13
**Diagnosis**				
Rheumatoid arthritis	Reference		Reference	
Healthy donors	- 7.33 (-11.38–3.27)	0.001	-7.05 (-10.74 to -3.35)	< 0.001
Ankylosing spondylitis	-6.23 (-11.31 - -1.16)	0.017	-6.94 (-11.69 to -2.18)	0.005
Early spondyloarthritis	-5.18 (-10.38 to -0.018)	0.05	-8.02 (-12.36 to -3.68)	< 0.001
Psoriatic arthritis	-8.36 (-13.76 to -2.96)	0.003	-7.69 (-12.34 to -3.05)	0.001

Coeff. Coefficient; CI. Confidence interva.l
